# Implementing a healthy postpartum lifestyle after gestational diabetes or preeclampsia: a qualitative study of the partner’s role

**DOI:** 10.1186/s12884-020-2769-6

**Published:** 2020-01-31

**Authors:** Ingfrid Almli, Hege S. Haugdahl, Heidi L. Sandsæter, Janet W. Rich-Edwards, Julie Horn

**Affiliations:** 1 Department of Obstetrics and Gynecology, Levanger Hospital, Nord-Trøndelag Hospital Trust, Levanger, Norway; 2 Department of Public Health and Nursing, Norwegian University of Science and Technology, Trondheim, Norway; 3 Department of Research, Nord-Trøndelag Hospital Trust, Levanger, Norway; 4 Department of Epidemiology, Harvard T.H. Chan School of Public Health, Boston, MA USA; 5 Division of Women’s Health, Department of Medicine, Brigham and Women’s Hospital and Harvard Medical School, Boston, MA USA

**Keywords:** Cardiovascular disease, Fathers, Gestational diabetes mellitus, Health behavior, Lifestyle, Maternal health, Partners, Preeclampsia, Qualitative study

## Abstract

**Background:**

Women with preeclampsia (PE) and gestational diabetes mellitus (GDM) are at increased risk for later cardiovascular disease, and lifestyle measures are recommended to prevent subsequent disease. Partner support has been shown to be important in lifestyle modification in other diseases, but there is a lack of knowledge of partner involvement in PE and GDM. The aim of this study was to explore the partner’s experiences and knowledge of gestational diseases, and how the partner wishes to contribute to lifestyle change.

**Methods:**

A qualitative study with one focus group interview and seven in-depth individual interviews, involving eleven partners of women with a pregnancy complicated by GDM or PE. The interview data were inductively analysed using four-step systematic text condensation, supported by interdependence theory.

**Results:**

Partners experienced a strong “we-feeling” and wanted to support the woman in lifestyle changes. At the same time, they felt insecure, worried, foolish and left out and they missed information from clinicians. The partners felt that their involvement was crucial to lasting lifestyle changes and expected that the clinicians would routinely invite them to discuss lifestyle change.

**Conclusions:**

Partners considered themselves an important resource for lifestyle changes for women with PE and GDM, but missed being more directly invited, informed and included in maternity care and wanted to participate in the care that followed the gestational disease. This study can help health professionals to realize that partners are an overlooked resource that can make important contributions to improve the health of the whole family if they are involved and supported by health services.

## Background

Preeclampsia (PE) and gestational diabetes mellitus (GDM) are common complications of pregnancy that are associated with increased risk of maternal type 2 diabetes (T2DM) [1, 2] and cardiovascular disease (CVD) later in life [3, 4]. The global prevalence of PE is around 5% [5], while in Norway it is stable at around 3% [6]. GDM prevalence varies globally in relation to race and ethnicity, ranging from 5.8% in Europe to 12.9% in Africa [7]. In Norway, the incidence of GDM has increased in recent years to around 5% of all pregnancies, partly due to new screening recommendations [8].

National and international guidelines recommend that a pregnancy complicated by GDM or PE should be followed by lifestyle changes such as increased physical activity, smoking cessation and healthy diet to prevent later CVD [8–11]. Pregnancy and new maternity may be a period when women have a desire to improve their lifestyle and is therefore referred to as a window of opportunity for lifestyle changes. During pregnancy, women have regular contact with health professionals who can provide them with information and support to change dietary and physical activity habits [12]. On the other hand, frequent breastfeeding, sleep deprivation and postnatal recovery can be so demanding that lifestyle changes are given low priority once the baby arrives [13, 14].

Previous qualitative studies have shown how women with complicated pregnancies consider partner support to be an important motivating factor to succeed in lifestyle changes in pregnancy or postpartum [15–17]. This is confirmed by a systematic review of behaviour theories that argues that social support can increase an individual’s capacity to change and retain new lifestyle habits [18].

The interdependence theory (IT) describes how the dyad, an exclusive relationship between two people, and the dyadic processes in a couple’s relationship will determine how the couple faces a health threat [19, 20]. IT focuses on the different structures of mutual dependence in a relationship. When health is threatened, a couple’s interdependence can transform motivation for change from being based on what is best for the individual to what is best for the relationship or partner.

Partner support has been shown to be a positive factor in other illness contexts [20–24]. However, to our knowledge, no previous study has focused on the role of the partner in lifestyle changes following PE and GDM. The purpose of this study was therefore to investigate the partner’s knowledge of the relationship between pregnancy complications and later risk of CVD, and to examine the factors that promote healthy lifestyle changes for the family during pregnancy and childcare.

## Methods

### Design

We chose a qualitative study design in order to explore opinions, attitudes, motives and experiences, where we gathered knowledge from focus group interviews and semi-structured in-depth individual interviews [25]. The dialogue in a focus group interview can promote a dynamic and informative exchange of thoughts and ideas on the topic of interest [26]. In individual semi-structured in-depth interviews, participants are given time and space to retrieve experiences and reflections that require a feeling of security to be shared [25].

### Participants

The informants were partners of women whose pregnancy involved GDM or PE and who gave birth between 2016 and 2018 in Levanger Hospital, Norway. It was important to elicit recent experiences to enable the partner to recall the stories, knowledge and questions that arose during the woman’s illness [27].

Partners had to be at least 18 years of age and able to understand and speak Norwegian. The partners were invited to prioritize diversity in terms of place of residence, number of children, ethnicity and type of pregnancy complication.

Diagnoses of PE and GDM were confirmed by medical record review. According to current national antenatal guidelines, GDM was defined as onset of glucose intolerance during pregnancy using a 75 g 2-h oral glucose tolerance test with fasting glucose levels of ≥5.3–6.9 mmol/l or a 2-h value of ≥9.0–11.0 mmol/l [8]. A diagnosis of PE required de novo hypertension (at least two blood pressure measurements of ≥140 mmHg systolic or ≥ 90 mmHg diastolic) after 20 weeks of gestation in combination with proteinuria of 1+ or more on a semiquantitative dipstick. PE complicated by severe hypertension (blood pressure greater than or equal to 160 mmHg systolic or greater than or equal to 110 mmHg diastolic) or with signs of significant end-organ dysfunction was considered severe PE [11].

### Recruitment

Retrospective recruitment was attempted by writing to 29 suitable women who had delivered between January 2015 and March 2017, while prospective recruitment involved a direct request to the maternity ward of the hospital for 35 deliveries from April to November 2018. Retrospective recruitment provided one response. Prospective recruitment was conducted by instructed midwives during the postnatal period in the ward. The researchers (IA, JH) validated the diagnosis before information letters were distributed. All relevant partners were invited to participate, and 14 of the 36 partners accepted. We offered flexibility regarding the time and place of the interview, but three of the participants still had to refuse because of work commitments, long journeys and other obstacles. Difficulties also arose in finding a suitable meeting time for focus groups. Only one focus group with four participants was therefore conducted, followed by individual in-depth interviews with seven individuals who did not participate in the focus group.

### Data collection

We developed a semi-structured interview guide (Additional file 1) to elicit partners’ experiences with a pregnancy complicated by PE or GDM and their knowledge of the association between these pregnancy complications and later risk of diabetes and CVD. In addition, we aimed to learn about motivation, barriers and facilitators to lifestyle modifications for the partner and his family. After two interviews, we customized the interview guide to enhance our understanding of the partners’ supportive role during pregnancy and his experience of lifestyle changes. The focus group lasted approximately 90 min and the semi-structured in-depth interviews from 45 to 90 min; all took place from June to December 2018. The focus group was supported by an experienced moderator (HSH). Interviews were conducted in the research department of the hospital, except for two that took place at other public institutions, and one that was conducted after working hours at the informant’s workplace. The interviews were digitally recorded and transcribed verbatim.

### Data analysis

Data were analysed using the four-step strategy of systematic text condensation and thematic cross-case analysis [28]. Coding and analysis were performed by the authors in cooperation, following these steps: 1) reading the interviews to gain an overall impression and find preliminary themes, 2) identifying and coding meaning units representing different aspects of participants’ experiences from pregnancy complications and lifestyle changes, 3) condensing the content of each of the coded groups, and 4) summarizing the content of each code group to generalized descriptions and concepts concerning partners’ coping with pregnancy complications. Analysis was data driven, although supported by interdependence theory [19, 20]. A codebook with dated reflections validated the analysis. The consolidated criteria for reporting qualitative research (COREQ) were used [29].

## Results

We collected data from eleven partners. Of these, five were partners of a woman diagnosed with PE, five of a woman with GDM, while one was the partner of a woman with a combination of PE and GDM. There was an even distribution between insulin- and diet-treated GDM and between mild and severe PE. The informants varied in age, place of residence, level of education and number of children (Table 1). One partner (informant) and two spouses were of non-European origin. The results of the analysis are presented in Table 2.

**Table 1:** Characteristics of study participants

	Age	Ethnicity (participant/woman)	Educational level	Employment	Place of residence	Pregnancy complication	Gestational length in weeks	Total number of children
Individual interviews								
Participant 1	30–34	Nordic/Nordic	Apprenticeship diploma	Full-time	Urban	Mild PE	≥37	1
Participant 2	25–29	Nordic/Nordic	Apprenticeship diploma	Full-time	Rural	Mild PE	≥37	1
Participant 3	25–29	Nordic/Nordic	Apprenticeship diploma	Full-time	Rural	Severe PE	34–36	2
Participant 4	35–39	Nordic/Asian	College/university	Full-time	Rural	Severe PE	< 34	1
Participant 5	40–44	Nordic/Nordic	Upper secondary school	Full-time	Urban	Severe PE and GDM	34–36	3
Participant 6	35–39	Nordic/Nordic	Upper secondary school	Full-time	Rural	GDM	≥37	2
Participant 7	35–39	Nordic/Nordic	College/university	Full-time	Rural	GDM	≥37	2
Focus group								
Participant 8	40–44	Middle East/Middle East	Apprenticeship diploma	Full-time	Urban	GDM	≥37	3
Participant 9	25–29	Nordic/Nordic	Upper secondary school	Full-time	Rural	GDM	≥37	3
Participant 10	30–34	Nordic/Nordic	College/university	Full-time	Urban	GDM	≥37	2
Participant 11	30–34	Nordic/Nordic	College/university	Full-time	Urban	GDM	≥37	1

*Abbreviations*: *PE* Preeclampsia, *GDM* Gestational diabetes mellitus

**Table 2:** Description of code groups and sub-codes

Code groups	Sub-codes
Feeling left out, insecure and worried, partners took it upon themselves to obtain information.	• Being right in the middle of things but without understanding what was going on • Uncertainty and worry about wife and child • Taking responsibility for the help they need
Partners learned about the gestational disease from the woman, but still felt a need for information from clinicians.	• Lack knowledge of later increased risk of CVD • Eye openers
Emotional and practical partner support – an investment for the whole family.	• We must do something about this together • I want to support and relieve her • Practical everyday help
Partners expected clinicians to invite them to discuss lifestyle changes.	• An overlooked resource • The mutual expectations of the partner and clinicians

### Feeling left out, insecure and worried, partners took it upon themselves to obtain information

Life with a partner with gestational disease affected the participants emotionally and practically, and they felt little involved in follow-up care. They felt that they had to take responsibility for getting the help they needed. They closely monitored the development of the disease, and felt stress and anxiety regarding the woman and baby. Even though they had attended pregnancy check-ups, they could feel left out, foolish and ignorant in interactions with friends or clinicians.

One partner had been worried about changes in the woman’s blood pressure and was stressed by all the check-ups and the thought that she could rapidly develop severe PE. When the woman was admitted to hospital, several partners experienced persistent stress, being prepared that the birth could come at any moment. The partners found they had little control and a poor overview of the situation, and did not understand why the clinicians could not tell them more about what the disease implied. One partner thought they were shielding the couple from information on how severe PE could be. They did understand that it could be difficult to predict how the disease would develop, but still felt that they should have received more detailed and consistent information about possible outcomes.

*“I felt that however high the levels of blood pressure and urine were, everything was fine … It all wore me out, and I can well understand that she got even more exhausted than me.”*

One partner was not informed of the diagnosis of severe PE until six weeks after the birth. The mother had received the information at a postnatal examination by her general practitioner. This partner struggled with the fact that he had not been told about the severity of her illness and its consequences at a much earlier stage. On the other hand, partners of women with mild PE had not felt great anxiety, although they did not understand why the woman became ill. They thought it happened just by chance. Lack of knowledge meant that some participants were confused and felt like outsiders in situations where the woman was referred several times by the local midwife to the hospital for check-ups.

The partners of women with GDM had varying knowledge of the disease. Some had never heard of GDM, including one who was afraid his wife would have diabetes for the rest of her life. They felt that the follow-up care was only aimed at the mother, and lacked appropriate information about the disease from health professionals to enable them to give her better support.

*“I knew nothing at all … I must say I’ve felt a bit stupid sometimes when people ask, ‘How’s that pregnancy diabetes going then?”*

In couples where one or both had qualifications in health care, the partners did not feel left out to the same extent because they already had some knowledge of the disease. Other partners felt uncertainty and a need for more knowledge, and did not understand why the woman did not receive more follow-up care from a doctor or a referral to a diabetes nurse.

Some were sceptical to blood pressure treatment, insulin treatment and other medication in pregnancy. They felt that it would be better to avoid such treatment.

Regular information that the baby was doing well was important for all partners, but partners of women with GDM were particularly concerned about the baby’s blood sugar level, birth weight and whether it could be born with diabetes.

Many partners obtained information on their own initiative to enable them to provide good support to the woman in pregnancy. Some searched in vain for information in the hospital, and many found information on the Internet. Several had accompanied the woman to pregnancy check-ups to learn more about the disease.

*“But we do see that there’s a lot of information that you don’t get hold of, and we discussed this after going to the hospital. Then I’d often heard one thing and she’d heard another thing. We understand more and find out the right way to interpret what was said when there’s a difference between the information the two of us got hold of. So it’s a good thing there are two of us …”.*

Others attended the check-ups because the woman was unfamiliar with the language and culture and because they had found that communication could be inadequate even if an interpreter was used. The women’s respect for authorities could mean that they did not ask clinicians various important questions.

The partners did not have the same opportunities as the pregnant women to contact health services, partly because they had to be at work. They found it very difficult to relate to the limited information that the doctor and other health professionals had given the woman.

### Partners learned about the gestational disease from the woman, but still felt a need for information from clinicians

Practical experience of the woman’s illness and symptoms provided partners with important lessons and reflections on what they could contribute themselves. In one couple where the woman had GDM, they both measured their blood sugar after eating the same food, which opened their eyes to the effect of starchy food. They found that her blood sugar level rose considerably and remained high for much longer than his. Another partner felt that he himself had perhaps learned the most from her GDM, because he discovered that the food he bought for the family contained too much natural sugar. Others experienced the benefits of physical exercise.

*“When my wife started to park the car 500 metres from her work and walked the last bit, we saw that that kept her blood sugar lower.”*

Apart from two who had studied healthcare, the partners were unfamiliar with the increased risk of CVD after PE or GMD, and did not believe that the woman had been given this information by clinicians. However, several partners of women with GDM knew of the increased risk of T2DM in later life.

There were different reactions to information on the increased risk of CVD. Some thought it that was just statistics, that illnesses cannot be controlled, and that a person can stay healthy and grow old anyway. Others were amazed that such important information had not been communicated already, since they had met many health professionals.

### Emotional and practical partner support – an investment for the whole family

A strong “we-feeling” was expressed in a number of ways even before the pregnancy complications were discovered. The partners attended pregnancy check-ups and supported the woman in any lifestyle changes she made.

*“The day we found out she was pregnant, we both stopped taking snuff.”*

Some couples had not discussed lifestyle at the beginning of the pregnancy, but those who did talk about it had generally agreed that they had a good lifestyle and did not need to change anything. Others had previously found that pressure from clinicians or family members regarding lifestyle changes would make the situation more difficult for the pregnant woman, and these partners supported the woman’s decision not to change her lifestyle in pregnancy, even though she was overweight.

Several of the partners of women with GDM expected the woman to take steps to control her blood sugar levels. None of the women with GDM had directly asked their partner to help them change their lifestyle. However, two partners found that the woman wanted to make changes of her own accord, and specifically stated that she did not need his support for this.

Although partners had expectations of themselves and wanted to help the woman make lifestyle changes when she was diagnosed with GDM, there was considerable variation in how they chose to support the woman. Some supported the woman by telling her to reduce sugar and fat in her diet, while others changed their own diet at the same time as the woman. Some participants found it difficult to change their lifestyle since they did not have diabetes themselves, but they did so on the grounds that it was easier for the woman and a good thing for the whole family. Others chose a halfway solution, being willing to eat the same dinner as the woman, but saw no need to change their breakfast and lunch habits if the couple did not eat together.

The partners assisted in a number of practical ways. The couples agreed to change their shopping habits from daily and partly spontaneous trips to the shops to planned purchases for a week. They became conscious of the importance of homemade food, often cooking dinner the previous evening, and heating it up the next day. They bought a freezer and always had healthy food available. Weekend sweets and soft drinks were replaced with fruit, vegetables and water. The families went out for walks more often.

Some partners found that the woman felt ashamed and had a guilty conscience and a feeling of failure because they were unable to control her blood sugar levels well enough with the lifestyle measures. The partners understood the woman’s emotional situation and felt that they had a sound basis for understanding the emotional and practical support the woman needed. Others chose to get involved by being more confrontational.

*“I was going to take out some rubbish. Then I noticed a chocolate wrapper on top of the rubbish. I asked my eldest boy ... No, he hadn’t eaten it. So then I had to take it up with my partner ... So then we found out that we’d better do something about this together. From that day on we cleared all the chocolate out of the cupboards and started again. So the fact that the partner also gets involved in the bit about changing the diet, I think in many cases that’s really vital for things to work.”*

The participants thought that the women might have managed the lifestyle changes alone during pregnancy, because they were highly motivated to stay healthy as possible so that their baby would be born healthy and normal. On the other hand, they felt that it would be difficult to continue the lifestyle changes after pregnancy without help.

*“As I said, I think it’s very difficult by yourself, I think it must be very difficult ... even if you have a support group, an app and a nutritionist, because they don’t come home with you and help you in the evenings, at weekends and in other situations where you might find it very hard to change your lifestyle.”*

The health benefits of lifestyle changes for the whole family were a major motivating factor for active participation in the woman’s change of diet. Partners who had experienced PE were positive towards helping to prevent CVD after pregnancy. Partners of women with severe PE reported being exhausted; the baby involved much work and they did not have the energy to start lifestyle changes until well after the birth. Some had previously experienced various obstacles to lifestyle changes; an example was that the woman preferred her childhood diet of mostly white rice and few vegetables. Others found that when the couple did not eat together for reasons such as night shifts, overtime and weekly commuting, both of them ate more unhealthy food. The partners spent a great deal of time with the mother and baby during the postnatal period. They helped to feed, change and look after the baby day and night, which gave the mother more energy to exercise.

*“Yes, I reckon I’m pretty good at helping her, she says so too ... There are two of us, so we both have to be ready to lend a hand.”*

However, factors such as the woman’s pelvic pain and too little sleep and energy during the postnatal period were found to constrain the partner’s efforts at lifestyle improvement.

*“Getting a baby in your house gives you some challenges in finding enough time ... So we might choose food that’s not quite so healthy. We’re a bit tired today: it’s easy just to order a pizza.”*

### Partners expected clinicians to invite them to discuss lifestyle changes

The partners felt that clinicians did not expect anything of them. None had received a written invitation from the health services to attend pregnancy consultations, except for routine ultrasound screening in the second trimester. However, they wanted to be invited when the consultation dealt with issues where their support for the woman could be important. This was especially true of partners of women with GDM; they wanted an invitation to learn more about how to help her change her lifestyle. The partners regarded themselves as an important resource and support for the woman.

*“They gave her a guilty feeling at the consultation in the first pregnancy, and because of that we thought, ‘That’s not how it’s supposed to be.’ That’s why in the second pregnancy we got involved early on and measured it ourselves, and told our GP to start insulin, and we used fast-acting insulin to control the fluctuations even better … That was because we knew so much ourselves, and we found we could take control of the treatment based on the knowledge we got in the first pregnancy.”*

The partners felt that all women who had had PE or GDM should have a postnatal consultation with their GP to receive information and follow-up care with the aim of avoiding illness in their next pregnancy and later in life. These expectations were expressed as follows:

*“There should be questions from the doctor about how you plan to continue in terms of lifestyle ... Not that you have to say it’s serious, but there are times in your life when you ought to do things, because we’re going to have another baby ... So it’s a good thing if we can avoid pregnancy poisoning and that stuff.”*

The participants agreed that information on the risk of disease had to be included in postpartum care. Brochures were often not read. The partners believed that all couples should be informed in early pregnancy that some pregnant women could develop GDM or PE, and how to attempt to prevent those diseases. However, some participants felt that pressure from the partner or clinician could give the woman stress and a negative self-image. They felt it was better if the couple found the right moment to make lifestyle changes themselves. Information was important because this would give the couple knowledge that they could use when they had more energy and time to begin lifestyle changes.

## Discussion

This study provides new knowledge of how partners of women with GDM and PE experience pregnancy complications and describes the partner’s role in lifestyle changes in a Scandinavian health care model. In line with studies of women with GDM and PE [17, 30, 31], the analysis showed that partners had little knowledge of gestational diseases and later risk of CVD, and sought knowledge about the diseases on their own initiative. The partners had a strong “we-feeling” and desire to help the women change their lifestyle. At the same time, they felt insecure, worried, left out and foolish. The results show that in situations where a woman is ill during pregnancy and childbirth, the partner needs information about her condition to give himself a sense of security and control. The partners wished that clinicians had involved them more during the pregnancy and expected to receive more information on how they could help to prevent subsequent CVD. Good partner knowledge is important in providing the necessary strength to deal with the emotional situation and the transition to fatherhood, while supporting the woman in her illness [32].

European studies of women who were overweight in pregnancy showed that 91% thought partner support and other social support were important in improving their lifestyle in pregnancy [15]. In qualitative studies of women with PE or GDM, the women state that their partner is important in supporting their lifestyle modification following gestational disease [16, 17, 33]. Nevertheless, our informants reported that the women had not directly asked their partners for support to change their lifestyle. Previous studies have reported that partner involvement in antenatal care is associated with positive health effects for the woman, her partner and their children [34, 35]. Including fathers in pregnancy and childbirth care is recommended as a health-promoting strategy, because it encourages the man to support the woman and enhances the couple’s communication and decision making concerning the health of mother and child [35]. Clinicians have a particular responsibility for enabling the woman’s partner to fulfil his role [32]. Our findings also reflect the clinical differences between the two pregnancy complications. Partners of women with GDM expected early involvement in lifestyle adaptation from mid-pregnancy, the usual time of diagnosis. In contrast, partners of women with PE required information and support to manage emotional distress associated with the acute onset of the disease before they could engage in healthy lifestyle changes.

Our study demonstrates a need for a greater focus on the partner by health professionals, as seen in previous research [34–37]. The Norwegian family health clinic was described by partners as being focused on mothers and children, making partners feel overlooked and excluded [37]. An African study showed that men need to understand what happens during pregnancy to be able to help the woman and child and therefore suggested that invitation letters and guidance should focus particularly on the woman’s partner [38]. Lifestyle changes in other contexts have shown that follow-up care adapted to both partners in a relationship can improve the health of both of them, and their relationship in general. The fact that partners were invited and involved by clinicians was of great importance for the couple’s health and lifestyle following obesity surgery [24] and in interventions to reduce the risk of CVD [21]. Partner’s understanding of the health risk is an important trigger for lifestyle changes [18, 22]. However, in their focus on delivering information to the individual, clinicians routinely neglect the impact of the partner in supporting behavior change and reducing conflict around lifestyle change [39]. Couple-based interventions are probably more effective than individual interventions if they strengthen the couple’s relationship and mutual interaction.

One of the principles of the interdependence theory of Lewis et al. is that couples can use different paths and actions to achieve the same goal of healthy living. This implies that interventions must be tailored to the individual couple. Health changes are easier to maintain if the couple experiences a mutual effect. The most important motivating factor for lifestyle changes for the partners in our study was good health for the whole family. Lewis et al. argue that lasting lifestyle changes are achieved in a number of ways: [1] the partners agree to perform the same health-promoting activities, [2] the partners agree on different activities/actions that one of them can perform, and [3] the couple agrees on different actions, one of which they can both perform [20]. The communication between the couple and the quality of their relationship will affect the goals they set for lifestyle change [20]. Our study does not include data on the quality of the couples’ communication, but the partners found that the couple set common goals. Table 3 shows examples of specific measures that illustrate how the couple performed individual and/or joint actions to achieve goals of lasting lifestyle changes. Collaborative behaviour is not only associated with the degree of health threat, but is also influenced by the couple’s ability to collaborate emotionally and reflectively on goals and measures. By strengthening these structures, couple-based lifestyle changes can become more permanent because the partners motivate each other and work together to reduce the health threat [20].

**Table 3:** Different approaches used by couples to achieve lasting lifestyle changes

Type of action	Examples from everyday life
The partners agree to perform the same health-promoting activities.	The partner changed his own diet at the same time as the woman, because that was easier for the woman and good for the whole family.
The partners agree on different activities/actions that one of them can perform.	The partner helped to feed, change and look after the baby day and night to give the mother more energy to exercise.
The partners agree on different actions where they could both perform one of them.	The partners agreed that it was important to be physically active but in different activities; one walked the dog and the other pushed the pram.

### Strengths and limitations

The informants in this study showed variation in age, occupation, number of children, place of residence and cultural background. This may have given us a broad idea of the thoughts and experiences of partners. However, selection bias could be perceived as a limitation of this study as partners that feel kept out and want to be more involved may be more likely to participate. The main purpose of qualitative research is to get deep knowledge about a topic and it was therefore crucial to recruit participants who were able to give thorough information about the research question and showed willingness to talk about it. Presentation of contextual background, such as demographic and study setting were offered so that readers are able to decide for which situations the findings might provide valid information [40]. The women’s diagnosis was systematically validated to exclude those with chronic diabetes or known hypertension. The focus group interview allowed informants to discuss, share thoughts and provide new input on comments by other group members. The in-depth interviews were adapted to the informants’ possibility to participate in terms of time and place, while the atmosphere in such interviews promotes trust and reflection, which may have resulted in more personal information than in a focus group setting. Except for one, the partners were interviewed within five months after the birth, thus minimizing the likelihood of recall bias.

There were only 11 informants, and a greater number might have enhanced the knowledge gained from the study. Including two diseases that develop quite differently could be a weakness because it may provide less knowledge about experiences related to each disease. However, recommendations for lifestyle change to prevent future CVD will be similar. Our aim was to explore a common approach to lifestyle changes for GDM and PE. Nevertheless, our findings regarding partners’ expectations of clinicians and health services may be of interest to health professionals in other countries in terms of increasing efforts to involve partners on the basis of the system in those countries.

## Conclusion

Our study shows that partners consider themselves to be an important resource in lifestyle changes for women with GDM or PE, but miss being more directly invited, informed and included in maternity care. Our results may help health services to direct their attention to an overlooked resource and give fathers a more active role in maternity and family care. This may be particularly important for women with pregnancy complications such as GDM or PE and can enhance their ability to make lasting lifestyle changes. Further research will show whether a greater focus on the partner and tailored couple-based interventions in maternity care can help reduce the risk of future illness for mothers and their families.

## Supplementary information

**Additional file 1.** Interview guide; English language version of the interview guide developed for the study.

## Data Availability

The interviews transcribed for the present study are not publicly available due to individual privacy considerations. Following possible approval by the Central Norway Regional Committee for Medical and Health Research Ethics, the corresponding author could make transcripts available upon reasonable request (with names redacted).

## Abbreviations

CVD: Cardiovascular disease
GDM: Gestational diabetes mellitus
IT: Interdependence theory
PE: Preeclampsia
T2DM: Type 2 diabetes
