# Supplementary material for: Implementing a healthy postpartum lifestyle after gestational diabetes or preeclampsia: a qualitative study of the partner’s role
Source: BMC Pregnancy Childbirth. 2020 Jan 31;20:66. doi: 10.1186/s12884-020-2769-6 (PMC6995219; doi:10.1186/s12884-020-2769-6)
Supplement: Supplementary file 1 — Additional file 1. Interview guide; English language version of the interview guide developed for the study. [file 12884_2020_2769_MOESM1_ESM.pdf]

Interview Guide

Start by mentioning that the moderator and secretary are legally bound to secrecy. (additional information for focus group: We would also like you, the participants, to agree on confidentiality within this group.)

It is up to each individual person how much sensitive information you want to share. Everything said here will be anonymized after the data has been processed. All the material will be stored in a locked cabinet.

There are no right or wrong answers. We are interested in your experiences, not so much general comments. We hope you can give some specific examples.

The interview starts with an open question, where the informants are asked to talk about the following:

1) How did you become involved when your partner got gestational diabetes/pre-eclampsia?

(The questions below can be elaborated by using sub-questions)

2) How have you experienced your role in general in during and after your partner's pregnancy?
    a. What information was given?
    b. Who informed/spoke to you and your partner?

3) Have you/you and your partner been informed about the relationship between gestational diabetes and/or pre-eclampsia and illness later in life? (If so, what information did you receive and from whom?)

Inform the participants about preeclampsia/gestational diabetes and future increased risk of cardiovascular disease and type 2 diabetes. Inform the participants that there is a good chance of preventing future CVD. It is an advantage that their risk was identified at an early stage. Diet, exercise, quitting smoking and monitoring of blood pressure and blood sugar are the most important preventive measures.

4) What do you feel about getting this information?

5) Is it important to get this information?

6) When would have been the best time to get this information?

Inform the participant about prevention of cardiovascular disease and women's need for partner support.

7) How do you see that you can help with lifestyle changes?
    a. Have you and your partner previously had any lifestyle change projects?
    b. How do you think your partner wants you to support her? Please give examples.
    c. How can a partner help without applying pressure? Are you afraid of putting pressure on her? Please elaborate.
    d. Do you have any examples of things you have done that positively or negatively influenced your partner's lifestyle?

8) What do you find prevents you and your partner from making lifestyle changes?

9) What kinds of help and support do you and your partner need, if any?
    a. What kind of support would you and your partner like in planning lifestyle changes and helping you to maintain them? (for example healthy life centre, doctor, health centre, individual programme on the Internet)

10) Researchers at the Norwegian University of Science and Technology are developing a lifestyle intervention program to promote lifestyle changes after pregnancy complicated by PE/GDM. This program will offer access to web-based lifestyle recommendations to reduce risk of cardiovascular disease. In addtion, a clinical dietician will provide individual advice and follow-up.
    a. Do you think that you would support your partner in participating in this program?
    b. Do you think partners should be included in the program?
    c. How do you think partners could be included?

11) Is there anything you would like to say or add that we haven't talked about much so far?
